# Machine Learning Meets Meta-Heuristics: Bald Eagle Search Optimization and Red Deer Optimization for Feature Selection in Type II Diabetes Diagnosis

**DOI:** 10.3390/bioengineering11080766

**Published:** 2024-07-29

**Authors:** Dinesh Chellappan, Harikumar Rajaguru

**Affiliations:** 1 Department of Electrical and Electronics Engineering, KPR Institute of Engineering and Technology, Coimbatore 641 407, Tamil Nadu, India; dinesh.chml@gmail.com; 2 Department of Electronics and Communication Engineering, Bannari Amman Institute of Technology, Sathyamangalam 638 401, Tamil Nadu, India

**Keywords:** classifiers, Bald Eagle Search Optimization, Red Deer Optimization, diabetes detection, performance analysis, feature extraction

## Abstract

This article investigates the effectiveness of feature extraction and selection techniques in enhancing the performance of classifier accuracy in Type II Diabetes Mellitus (DM) detection using microarray gene data. To address the inherent high dimensionality of the data, three feature extraction (FE) methods are used, namely Short-Time Fourier Transform (STFT), Ridge Regression (RR), and Pearson’s Correlation Coefficient (PCC). To further refine the data, meta-heuristic algorithms like Bald Eagle Search Optimization (BESO) and Red Deer Optimization (RDO) are utilized for feature selection. The performance of seven classification techniques, Non-Linear Regression—NLR, Linear Regression—LR, Gaussian Mixture Models—GMMs, Expectation Maximization—EM, Logistic Regression—LoR, Softmax Discriminant Classifier—SDC, and Support Vector Machine with Radial Basis Function kernel—SVM-RBF, are evaluated with and without feature selection. The analysis reveals that the combination of PCC with SVM-RBF achieved a promising accuracy of 92.85% even without feature selection. Notably, employing BESO with PCC and SVM-RBF maintained this high accuracy. However, the highest overall accuracy of 97.14% was achieved when RDO was used for feature selection alongside PCC and SVM-RBF. These findings highlight the potential of feature extraction and selection techniques, particularly RDO with PCC, in improving the accuracy of DM detection using microarray gene data.

## 1. Introduction

India faces a significant public health challenge with the escalating prevalence of diabetes. According to the International Diabetes Federation (IDF) Atlas data from 2021, a staggering 77 million adults in India already suffer from this chronic condition. Even more alarming are projections that this number will reach a critical mass of 134 million by 2045. This translates to approximately one in eight adults in India potentially having diabetes [1]. Particularly worrisome is the increasing number of young adults (below 40 years old) being diagnosed with type 2 diabetes.

Several factors contribute to this alarming rise. India’s rapid urbanization is linked to lifestyle changes that heighten diabetes risk. These changes include reduced physical activity levels, increased consumption of processed foods, and rising obesity rates. Additionally, genetic predisposition, stress, and environmental factors all play a role [2]. Unfortunately, many diabetes cases remain undiagnosed until complications arise, highlighting the need for increased awareness and early detection strategies.

The human cost of diabetes in India is immense, with this disease ranking as the seventh leading cause of death. The economic burden is equally concerning. It is estimated that diabetes drains the Indian economy of approximately USD 100 billion annually. Clearly, addressing this growing public health crisis requires a multipronged approach that focuses on preventive measures for type 2 diabetes, early diagnosis for all types, and proper management of the condition [3]. Considering these challenges, there is an urgent need for advanced diagnostic tools that can detect diabetes early and accurately. Traditional diagnostic methods, while useful, have limitations in terms of sensitivity and specificity. This has led researchers to explore innovative approaches, including the analysis of genetic data [4]. Microarray gene expression data have shown promise in identifying biomarkers associated with Type II Diabetes Mellitus (DM) [5]. However, the high-dimensional nature of microarray data presents its own set of challenges. The vast number of genes compared to the typically small sample sizes can lead to overfitting and reduced generalization in machine learning models [6]. This “curse of dimensionality” necessitates effective feature extraction and selection techniques to identify the most relevant genes for diabetes diagnosis.

In recent years, machine learning techniques have gained traction in medical diagnostics, offering the potential for more accurate and efficient disease detection. These methods, when combined with appropriate feature selection algorithms, can significantly enhance the accuracy of diabetes diagnosis. Meta-heuristic optimization algorithms have shown promise in feature selection tasks across various domains [7].

This study aims to address these challenges by investigating the effectiveness of feature extraction and selection techniques in improving the accuracy of Type II DM detection using microarray gene data. We explore three feature extraction methods: Short-Time Fourier Transform (STFT), Ridge Regression (RR), and Pearson’s Correlation Coefficient (PCC). Furthermore, we employ two meta-heuristic algorithms, Bald Eagle Search Optimization (BESO) and Red Deer Optimization (RDO), for feature selection. By combining these techniques with various classification methods, we seek to develop a robust framework for early and accurate diabetes detection.

### Review of Related Works

Early and accurate diagnosis of diabetes is critical for effective management, but traditional methods like blood glucose tests have limitations. Microarray gene technology offers a promising alternative by analyzing gene expression patterns in the pancreas, potentially revealing early signs of the disease [8]. Recent advancements in machine learning algorithms have further enhanced their effectiveness for diabetes detection. A novel Convolutional Neural Network (CNN) architecture was specifically designed for diabetes classification using electronic health records (EHR) data [9]. The model achieved an impressive accuracy of 85.2%, demonstrating the potential of deep learning for diabetes diagnosis from readily available clinical data. Explainable Artificial Intelligence (XAI) techniques with machine learning models for diabetes prediction were used [10]. The XAI approach not only achieved high accuracy but also provided insights into the factors most influencing model predictions. This interpretability is crucial for clinicians to understand and trust the model’s recommendations [11]. Certain investigations were made into the use of machine learning models that combine various data sources, including gene expression data, clinical information, and lifestyle factors. The findings suggest that integrating multimodal datasets can lead to more accurate and comprehensive diabetes risk prediction models [12]. Ensemble learning approaches continue to show strong performance. Specific algorithms like Random Forest and Deep Neural Networks have proven effective in various studies [13]. Microarray gene data analysis remains a valuable avenue for exploration. While traditional datasets have dominated research in machine learning for diabetes detection, microarray gene data offer a relatively unexplored avenue [14]. This presents an opportunity to investigate the effectiveness of various performance metrics such as accuracy, F1 Score, Mathew Correlation Coefficient, Jaccard Metrics, Error Rate and Kappa. 

Feature extraction and selection techniques play a crucial role in handling high-dimensional microarray data. Principal Component Analysis (PCA) was employed for dimensionality reduction in gene expression data, achieving improved classification accuracy for diabetes prediction [15]. Similarly, the utilization of Independent Component Analysis (ICA) for feature extraction demonstrated its effectiveness in identifying relevant gene signatures. Among feature selection methods, meta-heuristic algorithms have gained attention due to their ability to efficiently search large feature spaces. Particle Swarm Optimization (PSO) applied for feature selection in diabetes diagnosis showed improved accuracy and reduced Computational Complexity. Genetic Algorithms (GA) have also shown promise, as demonstrated by combining GA with Support Vector Machines for diabetes prediction using gene expression data [16]. Short-Time Fourier Transform (STFT) has been applied in various biomedical signal processing tasks, including ECG analysis for diabetes detection. However, its application to microarray data for diabetes diagnosis remains largely unexplored. Ridge Regression (RR) has been used for feature selection in high-dimensional biological data, but its potential in diabetes-specific gene expression analysis warrants further investigation.

PCC has been widely used for feature selection in bioinformatics, employed to identify diabetes-related genes from microarray data, achieving high classification accuracy. However, the combination of PCC with advanced meta-heuristic algorithms like Bald Eagle Search Optimization (BESO) and Red Deer Optimization (RDO) represents a novel approach in this field. Recent studies have highlighted the potential of BESO and RDO in various optimization problems. BESO applied to feature selection in cancer diagnosis demonstrated its effectiveness in handling high-dimensional data [17]. Similarly, RDO has shown promise in complex optimization tasks, as evidenced in protein structure prediction. However, the application of these algorithms to diabetes diagnosis using microarray data remains an open area of research. In terms of classification techniques, Support Vector Machines with Radial Basis Function kernel (SVM-RBF) have consistently shown strong performance in biomedical applications by achieving high accuracy using SVM-RBF for diabetes prediction from clinical data. The effectiveness of other classifiers such as Gaussian Mixture Models (GMMs), Expectation Maximization (EM), and Softmax Discriminant Classifier (SDC) in the context of diabetes diagnosis from gene expression data requires further exploration. While these studies have made significant contributions, there remains a need for comprehensive research that combines advanced feature extraction and selection techniques with state-of-the-art classification methods for diabetes diagnosis using microarray gene data. Table 1 represents the comparison of various machine learning approaches like various dimensionality reduction techniques and feature selection and classifiers of different datasets and accuracy.

## 2. Materials and Methods

**Figure 1:**
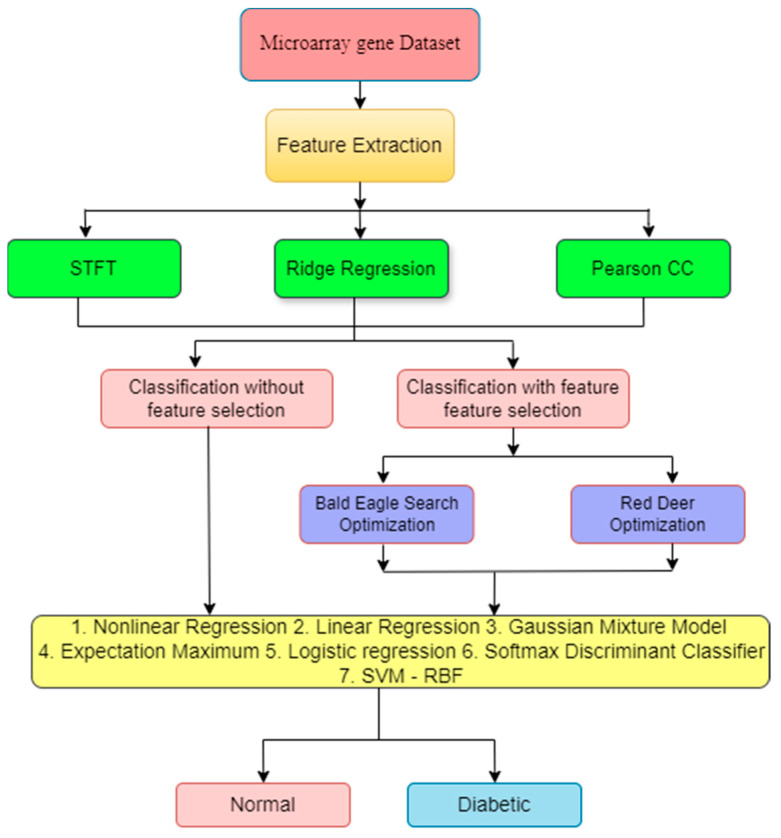
Overall flow diagram.

As depicted in Figure 1, this research utilizes a multistage approach for accurate diabetes detection. The first stage focuses on extracting relevant features from the microarray gene data. Three techniques are utilized for this purpose—STFT, RR, and PCC. The second stage involves two roots—in one, extracted data are directly used by classifiers to measure the performance; in the second, data are used in the feature selection process. In this research, FS used two meta-heuristic algorithms, BESO and RDO. These algorithms can help identify the most informative features from the initial extraction stage, potentially leading to a more efficient classification process. Finally, the third stage utilizes various classification techniques to categorize the data into diabetic and non-diabetic classes by using seven methods—NLR, LR, GMMs, EM, LoR, SDC, and SVM-RBF.

### 2.1. Dataset Details

Our research focused on utilizing microarray gene data from human pancreatic islets to detect diabetes and explore potential features associated with the disease. The data originated from the Nordic islet transplantation program (https://www.ncbi.nlm.nih.gov/bioproject/PRJNA178122). The data has accessed on 20 August 2021 and included gene expression profiles from 57 healthy individuals and 20 diabetic patients. To ensure data quality and facilitate analysis, preprocessing steps were implemented. First, genes with the highest peak intensity per patient were selected, resulting in a dataset of 22,960 genes. Next, a logarithmic transformation (base 10) was applied to standardize the data across samples, ensuring a mean of 0 and a variance of 1. This step helps to normalize gene expression values and account for potential variations between samples. Table 2 shows the details about the datasets used in this article. A key challenge in microarray data analysis is dealing with high dimensionality, referring to the large number of genes present. To address this, we employed feature extraction techniques. These techniques aim to reduce the number of features while retaining the most informative content relevant to diabetes detection [30]. Following feature extraction, feature selection techniques were applied to further refine the dataset and potentially improve classification accuracy. Specifically, two optimization algorithms were utilized, BESO [31] and RDO [32]. These algorithms helped identify the most relevant features for diabetes detection, further reducing the dimensionality of the data. Finally, to evaluate the performance and accuracy of diabetes classification, seven different classification algorithms were used. These algorithms will be discussed in detail later in this article.

### 2.2. Need for Feature Extraction (FE)

Microarray data analysis presents a challenge due to its high dimensionality. This means the data include a vast number of genes, which can be overwhelming for classification algorithms. A high number of features can lead to a phenomenon called the “curse of dimensionality”. This occurs when the number of features [33] grows exponentially, making it difficult for classification algorithms to learn effective decision boundaries and potentially leading to overfitting. Feature extraction techniques help address this challenge by reducing the number of features while retaining the most informative ones for diabetes detection. This process essentially focuses the analysis on the most relevant aspects of the gene expression data that contribute to differentiating between diabetic and non-diabetic samples. FE acts as a filter, selecting the most informative features from the vast amount of gene expression data. This allows the classification algorithms to work with a more manageable dataset and ultimately leads to more accurate diabetes detection.

#### 2.2.1. Short-Time Fourier Transform (STFT) 

STFT offers a valuable technique for analyzing microarray gene expression data. It excels at capturing frequency domain information within specific time windows. This allows researchers to condense the dataset by extracting key features that represent how gene expression levels change over time and across various frequencies. Like its application in QRS complex detection by [34], STFT provides a time–frequency representation of gene expression data. This visualization facilitates exploration of how gene expression levels fluctuate across time and different frequency components. A key strength of STFT lies in its ability to pinpoint specific time intervals where frequencies are dominant. This allows researchers to identify genes with temporal patterns that activate during distinct time periods within the data. Additionally, STFT contributes to dimensionality reduction by extracting significant genes or groups of genes associated with specific frequency components [35]. This targeted approach streamlines the exploration of gene expression dynamics, ultimately aiding in the discovery of biologically relevant patterns within the data.

(1) $$X\left[m,w\right]=\sum_{n=-\infty }^{\infty }{x\left[n\right]w\left[n-m\right]e}^{-iwn}$$

where $x\left[n\right]$ is the input data, and length n is 0, 1, 2, …, n − 1; $w\left[m\right]$ is the representation for the STFT window and is 0, 1, 2, …, m − 1. Moreover, $i=\sqrt{\left(-1\right)}$ is the complex variable. 

#### 2.2.2. Ridge Regression (RR)

The analysis begins by focusing on smaller groups of samples. For each group, a feature matrix ($X_{i}$) is created. This matrix includes information about each sample as data point in a row, along with its corresponding outcome in an outcome vector ($Y_{i}$) [36]. Ridge Regression is then applied to this local data to identify patterns. The estimates obtained by applying Ridge Regression to these local groups are referred to as local Ridge Regression estimates.

(2) $${\hat{\beta }}_{i}={\left(X_{i}^{T}X_{i}+\lambda_{i}I_{p}\right)}^{-1}X_{i}^{T}Y_{i}$$

(3) $${\hat{\beta }}_{dist}=\sum_{i=1}^{q}\left(\omega_{i}{\hat{\beta }}_{i}\right)$$

Analyzing the estimation error in distributed Ridge Regression benefits from studying it within the framework of finite sample analysis for linear models. In this context, we consider the standard linear model, where Y represents the outcome variable, X is the feature matrix, b is the coefficient vector, and ε represents the error term. The outcome for each of the ‘n’ independent samples is represented by $Y\;\in \;R^{n}$ a continuous vector with ‘n’ dimensions. This study utilizes a data matrix, X, to represent the features of the samples. This matrix has dimensions n × p, where ‘n’ represents the number of samples and ‘p’ represents the number of features measured for each sample. We are also interested in a vector of coefficients, denoted by beta (β). $\beta \;=\;{\left(\beta_{1},\;\beta_{2}{,\;\beta }_{3},\;\ldots ,\;\beta_{p}\right)}^{T}\;\in \;R^{n}$ This vector has ‘p’ dimensions and contains unknown values, which aim to estimate through analysis. These coefficients will ultimately reveal how much each feature contributes to the outcome variable we are trying to predict. This technique is employed for two main purposes: predicting the outcome variable for new samples and accurately estimating the influence of each feature (coefficient) on that outcome. However, it is important to consider that random errors or noise can significantly impact the outcome vector, $\varepsilon \;=\;{\left(\varepsilon_{1},\;\varepsilon_{2},\;\ldots ,\;\varepsilon_{n}\right)}^{T\;}\in \;R^{n}$, which represents the actual values that are trying to predict [37].

In linear models, we often assume that the errors in the outcome variable (represented by the vector ε) are independent of each other, have an average value of zero, and a constant variance $\sigma^{2}$. Ridge Regression is a popular technique for estimating the coefficients (β). Here, the equation for the same is:

(4) $$\hat{\beta }\;(\lambda )\;=\;{\left(X^{T}X\;+\;n\lambda I_{p}\right)}^{-1}X^{T}Y$$

The Ridge Regression technique incorporates a parameter known as lambda (λ), which plays a crucial role in tuning the model. This parameter offers several advantages when it comes to estimating the coefficients (β). One key benefit is that it helps to ‘shrink’ the coefficients obtained from Ordinary Least Squares regression, often leading to improved estimation accuracy. Now, let us consider a scenario where our data samples are spread across multiple locations or machines, represented by ‘q’ in total. In this case, we can partition the data for analysis as:

(5) $$X=\left[\begin{array}{c} X_{1}\\ \vdots \\ X_{q} \end{array}\right]\;and\;Y=\left[\begin{array}{c} Y_{1}\\ \vdots \\ Y_{q} \end{array}\right];$$

In distributed computing environments, where data are spread across multiple machines, approximate solutions for Ridge Regression are often preferred. This approach involves performing Ridge Regression locally on smaller subsets of the data ($X_{i},\;Y_{i})$ at each machine [38]. To achieve this, local Ridge Regression with a regularization parameter $\lambda_{i}$ is performed on each data subset ($X_{i},\;Y_{i})$. The formula for these local ridge estimators is as follows:

(6) $${\hat{\beta }}_{i}(\lambda_{i})={\left(X_{i}^{T}X_{i}+n_{i}\lambda_{i}I_{p}\right)}^{-1}X_{i}^{T}Y_{i}$$

Here,

- ${\hat{\beta }}_{i}$ represents the locally estimated coefficient vector for a specific data subset.
- $X_{i}$ denotes the design matrix for the i-th data subset.
- $Y_{i}$ represents the outcome vector for the i-th data subset.
- $\lambda_{i}$ is the regularization parameter for the local Ridge Regression on the i-th subset.
- I is the identity matrix with the same dimension as $X_{i}^{T}X_{i}$.

By using a weighted one-shot distributed estimation summation, the local ridge estimators from each data subset are combined as:

(7) $${\hat{\beta }}_{distributed}=\sum_{i=1}^{q}\left(\omega_{i}{\hat{\beta }}_{i}\right)$$

where

- ${\hat{\beta }}_{distributed}\;$ represents the final combined coefficient vector obtained from all data subsets.
- $\omega_{i}$ represents the weight assigned to the local estimator from the i-th data subset.
- ${\hat{\beta }}_{i}$ represents the locally estimated coefficient vector for the i-th data subset (as defined earlier).

Unlike Ordinary Least Squares (OLS), the local Ridge Regression estimators we defined previously have some inherent bias. Due to this bias, there is no effect of imposing constraints on the weights used to combine them. This approach is particularly well suited for data matrices (X) with any covariance structure (Σ). Assuming we have ‘n’ samples that are equally distributed across different machines, we can compute a local ridge estimator (${\hat{\beta }}_{1}$) for each data subset [39]. Additionally, we can estimate local values for the signal-to-noise ratio (SNR, represented by ${\hat{\sigma }}_{i}^{2}$ and the noise level ${\hat{a}}_{i}^{2}$ for each subset). To find the optimal weights for combining these local estimators, we consider three key factors: m, m0, and λ.

#### 2.2.3. Pearson’s Correlation Coefficient (PCC)

PCC [40] serves as a powerful statistical tool for analyzing microarray gene expression data in diabetes detection. It measures the strength and direction of the linear relationship between gene expression levels. High PCC values, positive or negative, indicate that gene expression levels tend to fluctuate together. This co-fluctuation can reveal underlying biological processes. Genes with strong positive PCC may be regulated by similar mechanisms, potentially impacting diabetes development. Conversely, genes with strong negative PCC might play opposing roles in cellular processes, with one potentially protective against diabetes while the other linked to disease progression.

By analyzing PCC values between gene pairs, we can identify potentially valuable genes for diabetes detection. Genes with strong positive correlations may act together to influence the diabetic state. Conversely, genes with strong negative correlations might represent opposing pathways with implications for disease development [41].

PCC analysis helps us understand how gene expression levels co-vary, ultimately aiding in the selection of informative gene sets that can be used to build robust models for accurate diabetes classification.

(8) $$\rho \left(x,y\right)=\frac{cov(x,y)}{\sigma_{x}\sigma_{y}}$$

(9) $$cov\left(x,y\right)=\frac{\sum_{i=1}^{n}\left(x_{i}-\bar{x}\right)(y_{i}-\bar{y})}{n-1}$$

cov(x,y) indicates the covariances between x and y.

(10) $$\sigma_{x}=\sqrt{\frac{\sum_{i=1}^{n}{\left(x_{i}-\bar{x}\right)}^{2}}{n-1}}$$

(11) $$\sigma_{y}=\sqrt{\frac{\sum_{i=1}^{n}{\left(y_{i}-\bar{y}\right)}^{2}}{n-1}}$$

$\sigma_{x},\;\sigma_{y}$ indicated the Standard Deviation of x and y.

From Table 3, analyzing various features extracted from the data, STFT and RR show minimal class differences with a mean of 40.7 and a variance of approximately 11,700 between diabetic and non-diabetic groups, suggesting they might not be strong discriminators for diabetes. Slight variations in Skewness and Kurtosis for most features hint at some distributional differences between the classes. Sample and Shannon Entropy values are identical within each class but differ between them, potentially indicating distinct underlying data patterns. Higuchi’s Fractal Dimension [42], however, stands out with a clear distinction (approximately 1.1 for STFT and 2.0 for diabetic class data) between classes for all features, suggesting its potential value in diabetes detection. Finally, Canonical Correlation Analysis (CCA) reveals a significant difference between diabetic and non-diabetic classes for the diabetic class data (0.4031 vs. 0.0675). This highlights CCA’s potential to identify features within the diabetic class data that are relevant for diabetes classification [43]. Overall, the table emphasizes the importance of analyzing various statistical parameters to understand the characteristics of different features and their potential role in differentiating diabetic and non-diabetic samples. The observed variations in Skewness and Kurtosis suggest that the data may not follow a typical Gaussian (normal) distribution and might exhibit non-linear relationships. This can be further confirmed by visualizing the data using techniques like histograms, Normal Probability plots, and scatter plots of the feature extraction outputs.

**Figure 2:**
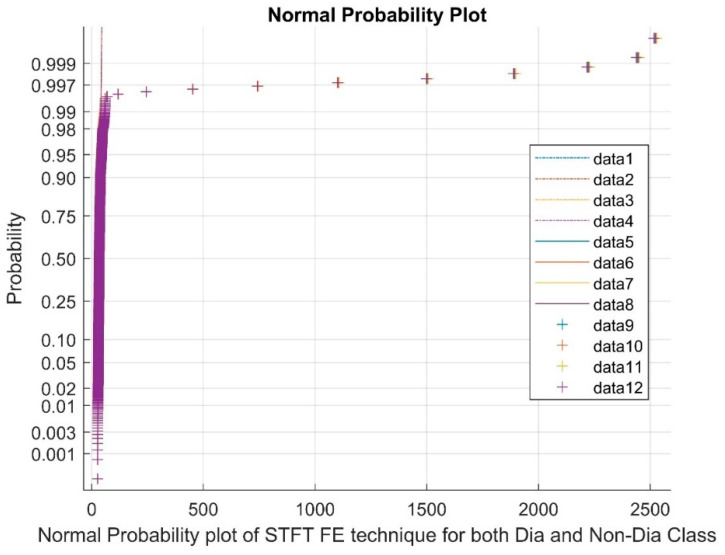
Normal plot of STFT FE techniques for both diabetic and non-diabetic class.

Figure 2 shows the Normal Probability plot of STFT for both diabetic and non-diabetic class, from the data points 1 to 4 as reference points, from 5 to 8 as upper bounds and from 9 to 12 as clustered variable points.

**Figure 3:**
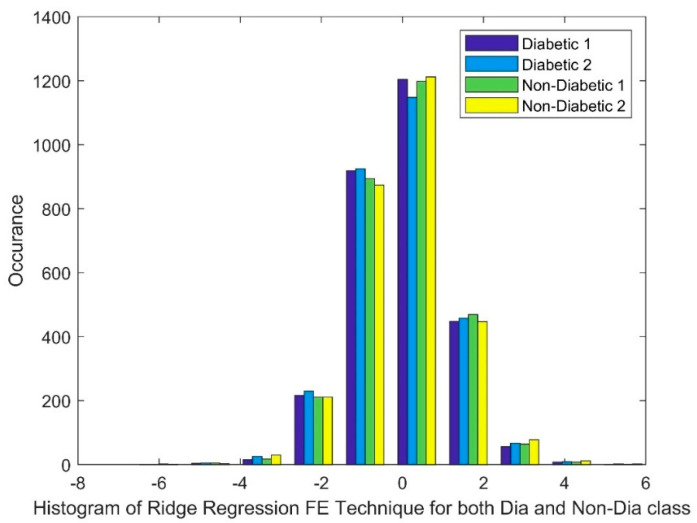
Histogram of RR FE techniques for both diabetic and non-diabetic class.

Figure 3 shows the histograms of the RR FE technique applied to both diabetic and non-diabetic classes. While the distributions appear somewhat bell shaped, suggesting a possible tendency towards normality, a closer look reveals some deviations. Notably, there is an overlap between the distributions of the diabetic and non-diabetic classes. This overlap, along with potential deviations within each class distribution, suggests a more complex, non-linear relationship between the classes.

**Figure 4:**
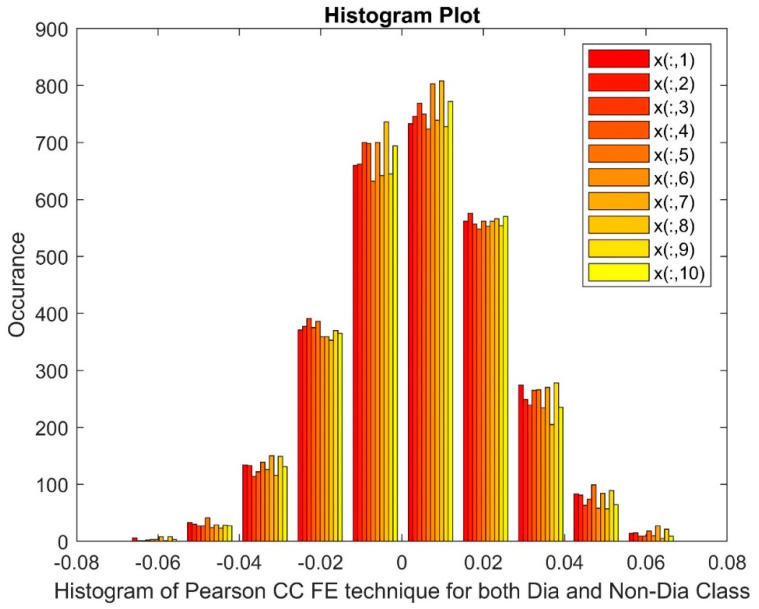
Histogram of PCC FE techniques for both diabetic and non-diabetic class.

Figure 4 displays histogram of the PCC FE technique applied to both diabetic and non-diabetic classes. The markers on the x-axis represent patients, with x(:,1) indicating patient 1 and x(:,10) indicating patient 10. The histograms reveal a skewed distribution of values, suggesting a non-normal data pattern. Additionally, a gap and potential non-linearity are observed in the data distribution for this method.

Fron all the techniques of FE, it is revealed that non-gaussian, non-linearity and techniques highlight the complexity of the data observed across the classes and the potential for further feature selection is essential.

## 3. Feature Selection Method

As mentioned in Section 1, there are two meta-heuristic algorithms, namely BESO and RDO, used as feature selection techniques that should be compared with and without the FS method from FE techniques to classifiers to enhance classifier performance.

### 3.1. Bald Eagle Search Optimization (BESO)

Bald Eagle Search Optimization (BESO) is a recent addition to the field of meta-heuristic algorithms. It draws inspiration from the hunting behavior of bald eagles, specifically their adeptness in locating and capturing prey. During the optimization process, BESO utilizes various search strategies modeled on these predatory behaviors. These strategies allow the search agents to explore the search space effectively, ultimately leading to the identification of optimal solutions for complex real-world problems.

- Step 1: Simulating Exploration: Selecting the Search Area

The BESO algorithm mimics the exploration phase of a bald eagle’s hunt through its first stage. In this stage, the algorithm determines a suitable search area for potential solutions, like how a bald eagle might scout a vast region for prey, which is mathematically stated as

(12) $$P_{new}^{t}=P_{Best}^{t}+ar(P_{mean}^{t}-P_{i}^{t})$$

A control parameter (a) set between 1.5 and 2 guides the search area’s size, while a random number (r) between 0 and 1 introduces an element of chance, preventing stagnation in local optima. This exploration is further influenced by two key factors. The algorithm considers the best solution found so far ${(P}_{Best}^{t})$ to guide its search towards promising regions. Additionally, the average position of the current eagle population $\left(P_{mean}^{t}\right)$ is factored in to ensure exploration extends beyond the immediate vicinity of the best solution and delves into new areas.

- Step 2: Intensifying the Search

The second stage of BESO intensifies the search for optimal solutions within the exploration area identified in phase one. This mimics the behavior of a bald eagle that has located a promising hunting ground. Here, the search agents meticulously scan the defined region. They follow a spiral-like pattern, like how a bald eagle might meticulously search a lake for fish. This focused search pattern allows the algorithm to efficiently explore the selected area and identify potential solutions with greater precision. The mathematically expression as:

(13) $$P_{new}^{t}=P_{i}^{t}+y_{i}X(P_{i}^{t}-P_{i+1}^{t})+x_{i}X(P_{i}^{t}-P_{mean}^{t})$$

where

$$\begin{array}{c} x_{i}=\frac{xr_{i}}{max\left|xr\right|},\;y_{i}=\frac{yr_{i}}{max\left|yr\right|}\\ xr_{i}=r_{i}\;X\;sin\left(\theta_{i}\right),\;yr_{i}=r_{i}\;X\;cos(\theta_{i})\\ \theta_{i}=a\;X\;\pi \;X\;rand,\;a\in \left(5,10\right)\\ r_{i}=\theta_{i}+R+rand,\;R\in [0.5,2] \end{array}$$

The two random parameters, denoted as ‘a’ and ‘R’, control the search pattern within the chosen exploration area. These parameters influence the number of spirals undertaken by the search agents and the variation in their coiling shape. This element of randomness helps prevent the algorithm from becoming stuck in suboptimal solutions. By introducing variation in the search pattern, BESO encourages exploration of diverse positions within the search area, ultimately leading to the identification of more accurate solutions.

- Step 3: Convergence

The final stage of BESO, represents the convergence towards the optimal solution [44]. In this stage, the search agents gravitate towards the most promising position identified so far. This convergence process can be mathematically written as:

(14) $$P_{new}^{t}=rand\;X\;P_{Best}^{t}+{x1}_{i}\;X\;(P_{i}^{t}-C_{1}\;X\;P_{mean}^{t})+{y1}_{i}\;X\;(P_{i}^{t}-C_{2}\;X\;P_{Best}^{t})$$

where

$$\begin{array}{c} {x1}_{i}=\frac{xr_{i}}{max\left|xr\right|},{y1}_{i}=\frac{yr_{i}}{max\left|yr\right|}\\ xr_{i}=r_{i}\;X\;sinh(\theta_{i}),\;yr_{i}=r_{i}\;X\;cosh(\theta_{i})\\ r_{i}=\theta_{i},\;\theta_{i}=a\;X\;\pi \;X\;rand,\;a\in (5,10) \end{array}$$

Two random parameters ($C_{1},\;C_{2}$) play a crucial role in this stage. These parameters, ranging between 1 and 2, control the intensity of the search agents’ movement towards the best solution.

### 3.2. Red Deer Optimization (RDO)

Red Deer Optimization (RDO) is a recent metaheuristic algorithm inspired by the fascinating mating behavior of red deer during the rut, or breeding season. Introduced in 2016 [45], RDO mimics this natural process to find optimal solutions to complex problems. The algorithm starts with a random population of individuals, representing potential solutions to the diabetes detection problem. These individuals are analogous to red deer. The strongest individuals, like dominant males, are identified. These “stags” compete for influence over the remaining population, like how stags fight for harems of females during the rut. The outcome of this competition determines how solutions are combined to create new offspring, representing the next generation of potential solutions. Stronger “stags” have a greater chance of mating with more “hinds”, leading to a wider exploration of the solution space. Weaker individuals may still contribute, but to a lower extent. This process balances exploration and exploitation [46]—key aspects of optimization. Through this iterative process of selection, competition, and reproduction, RDO progressively refines the population towards improved solutions for diabetes detection. The final population contains the most promising solutions identified by the algorithm [47].

## 4. The RDO Algorithm

(a) Initialization: Each RD is described by a set of variables, analogous to its genes. The number of variables (XNvar) corresponds to the number of genes. The values of these variables represent the potential contribution of each gene to diabetes detection, for instance, the set XNvar to 50, which means to account for investigations.

Initializing Individual Positions:

The position of each RD (Xi) is defined by this set of variables. Here, Xi would be a vector containing the values for each of the 50 genes (e.g., [θ1, θ2, …, θ50]). These initial values are assigned randomly.

(b) Roaring: In the “roar stage”, each RD representing a potential gene selection for diabetes detection can explore its surrounding solution space based on the microarray gene data. Imagine each RD has neighboring solutions in this multidimensional space. Here, a RD can adjust its position, which means gene selection within this local area. The algorithm evaluates the fitness of both the original and the adjusted positions using a fitness function reflecting how well the gene selection differentiates between diabetic and non-diabetic cases.

An equation is used to update the RD positions,

(15) $${Male}_{new}=\left\{\begin{array}{c} {male}_{new}+a_{1}\times (((UB-LB)\times a_{2})+LB,ifa_{3}\geq 0.5\\ {male}_{new}-a_{1}\times (((UB-LB)\times a_{2})+LB,ifa_{3}<0.5 \end{array}\right\}$$

Here, a_1_, a_2_, and a_3_ are the random factors between 0 and 1. ${Male}_{old}$ and ${Male}_{new}$ are the old and new positions of RDs. This process is akin to the RD exploring its surroundings and “roaring” if it finds a better location with a higher fitness value (B*) [48]. Such successful adjustments elevate the RD to commander status, signifying a promising solution for diabetes detection. Conversely, if the exploration yields a position (A*) with a lower fitness value compared to the original, the Red Deer remains a “stag”. These stags still have the chance to contribute in later stages of the algorithm. This roar stage essentially refines the initial population by focusing on the RD gene selections that demonstrate better potential for differentiating diabetic and non-diabetic cases based on the gene data.

(c) Roar:

Following the roar stage, commanders engage in a unique form of competition with the remaining stags. This competition does not involve literal combat, but rather an exchange of information about their gene selections.

(16) $${New}_{1}=\frac{\left(Com+Stag\right)}{2}+b_{1}\times (((UB-LB)\times b_{2})+LB)$$

(17) $${New}_{2}=\frac{\left(Com+Stag\right)}{2}-b_{1}\times (((UB-LB)\times b_{2})+LB)$$

Each commander interacts with a set of randomly chosen stags. The fight’s outcome determines how much information, or details about their gene selections, the commander shares with the stags. Commanders with demonstrably better gene selections will share more information with the stags they compete with. Imagine commanders with promising solutions guiding the stags towards improvement. Here, commanders with demonstrably superior gene selections retain their position as leaders and share more knowledge with the stags. Conversely, if a stag’s gene selection shows potential during the competition, it might surpass the commander’s current selection. In such cases, the stag’s improved solution becomes the new leader, and the information flow is reversed.

(d) Creation phase of harems:

Following the roar and fight stages, commanders are rewarded based on their performance. This reward system is reflected in the formation of harems. A harem consists of a commander and a group of hinds as female deer. The number of hinds a commander attracts is directly proportional to his success in the previous stages. Commanders with demonstrably superior gene selections will attract larger harems. Imagine a commander’s “power” being determined by how well its gene selection differentiates between diabetic and non-diabetic cases.

(18) $$N\;{harem}_{n}=\mathrm{round}\;\{p_{n}\;\times \;N_{hind}\}$$

Commanders with greater power will attract more hinds to their harems. Stags, on the other hand, are not included in harems. This reward system incentivizes the continued exploration and refinement of promising gene selections for diabetes detection.

(e) The mating phase in RDO occurs in three key scenarios: 1. Commander Mating Within Harems—Each commander has the opportunity to mate with a specific proportion (α) of the hinds within its harem. This mating metaphorically represents the creation of new gene selections based on combinations of the commander’s strong selection and those of the hinds. 2. Commander Expansion Beyond Harems—Commanders can potentially mate with hinds from other harems. A random harem is chosen, and its commander has the chance to “mate” with a certain percentage (β) (which lies between 0 and 1) of the hinds in other harems. 3. Stag Mating—Stags also have a chance to contribute. They can mate with the closest hind, regardless of harem boundaries. This allows even less successful gene selections to potentially contribute to the next generation, introducing some level of diversity. By incorporating these three mating scenarios, RDO explores a combination of promising gene selections, ventures beyond established solutions, and maintains some level of diversity through stag mating.
(f) Mating phase—New solutions are created. This process combines the strengths of existing gene selections from commanders, hinds, and even stags, promoting a balance between inheritance and exploration. This approach helps refine the population towards more effective gene selections for diabetes detection using your microarray gene data.
(g) Building the next generation—RDO employs a two-pronged approach to select individuals for the next generation. A portion of the strongest RD is automatically selected for the next generation. These individuals represent the most promising gene selections identified so far. Additional members for the next generation are chosen from the remaining hinds and the newly generated offspring. This selection process often utilizes techniques like fitness tournaments or roulette wheels, which favor individuals with better fitness values.
(h) RDO’s stopping criterion to determine the number of Iterations—a set number of iterations can be predetermined as the stopping point. The algorithm might stop if it identifies a solution that surpasses a certain threshold of quality for differentiating diabetic and non-diabetic segregation. A time limit might also be set as the stopping criterion. The parameters of each value involved in this algorithm are described in Table 4.

Based on the *p*-value selection criteria employed in Table 5, features extracted using STFT, RR, and PCC might not be the most informative for diabetes detection. This conclusion is drawn from analyzing *p*-values obtained through *t*-tests for various FE methods for two FS techniques as BESO and RDO were revealed that *p*-values can serve as initial indicators to quantify the potential presence of outliers, non-linearity, and non-Gaussian data distributions within the classes. Further analysis may be necessary to identify the most statistically significant features for accurate diabetes classification.

### Analyzing the Impact of Feature Extraction Methods Using Statistical Measures

**Figure 5:**
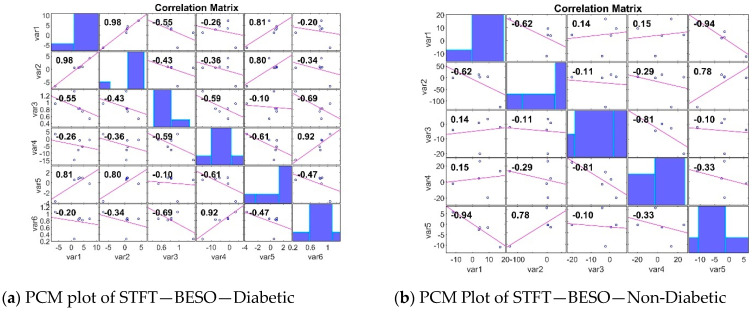
PCM plots for the STFT FE method.

**Figure 6:**
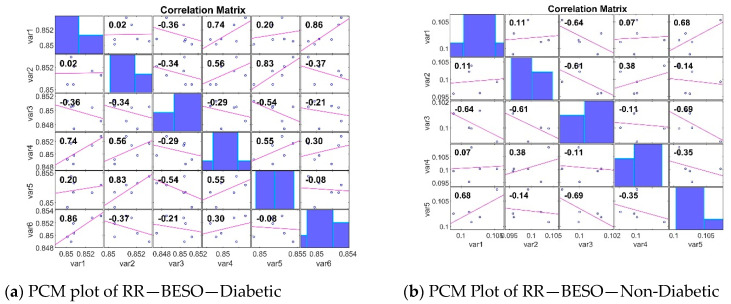
PCM Plots for the RR FE method.

The PCM analyses of gene expression in diabetic and non-diabetic patients reveal crucial insights into the relationships between different genes. Diabetic patients show a broader range of strong positive and negative correlations, indicating clusters of co-expressed genes and genes with opposing expression patterns. In contrast, non-diabetic patients exhibit weaker correlations and a more diverse expression pattern. Identifying these patterns is essential for understanding the underlying mechanisms of diabetes and for discovering potential biomarkers for diagnosis and treatment. The analysis of the Pearson Correlation Matrix (PCM) [49] in both diabetic and non-diabetic patients reveals significant insights into gene expression patterns. In diabetic patients, the STFT BESO values range from −0.69 to 0.98, with strong positive correlations such as 0.98 between var1 and var2, and negative correlations like −0.69 between var3 and var6, indicating clusters of co-expressed genes and opposing expression patterns. For non-diabetic patients, the RR BESO values range from −0.94 to 0.78, showing weaker correlations overall, with notable positive correlations such as 0.68 between var1 and var5, and negative correlations like −0.64 between var1 and var3, suggesting a more diverse gene expression pattern (Figure 5). Another PCM analysis for non-diabetic patients shows values ranging from −0.54 to 0.86, with significant correlations including a strong 0.83 between var2 and var5, and a negative −0.34 between var2 and var3, providing a baseline for comparison. An additional PCM for diabetic patients indicates values from 0.11 to 0.68, with a notable positive 0.92 between var4 and var6, and a negative −0.47 between var5 and var6, reinforcing the presence of significant gene clusters (Figure 6).

## 5. Classifiers

FE acts like a filter, selecting the most informative data tidbits from the massive count of gene expressions. However, these informative features alone are not enough to diagnose diabetes. Here is where classifiers come in as powerful tools. They analyze the selected features, learning the subtle patterns that differentiate diabetic from non-diabetic samples. These patterns can be complex and non-linear, making them difficult to identify by hand. Classifiers ultimately leverage this knowledge to make automated and accurate classifications of new, unseen diabetic cases.

### 5.1. Non-Linear Regression

While standard Linear Regression assumes a straightforward, linear relationship between variables, Non-Linear Regression emerges as a powerful tool for diabetes detection. It tackles the challenge of complex relationships that might exist between features extracted from gene expression data and the presence of diabetes. The core principle involves modeling the relationship between extracted features and the diabetic state using a specific mathematical function that captures the non-linearity [50]. The model strives to minimize the sum of squared errors between the predicted diabetic state and the actual diabetic state for each data point. Imagine finding the best-fitting non-linear curve that represents the data.

Least squares estimation then comes into play to determine the unknown parameters within the chosen non-linear function. These parameters define the shape and characteristics of this non-linear relationship. After training the model with known diabetic and non-diabetic data points, the estimated non-linear function can predict the diabetic state for new, unseen cases based on their extracted features. A threshold probability or value can be established to classify the predicted state as diabetic or non-diabetic. This approach offers a valuable technique for leveraging the complexities within gene expression data for improved diabetes detection.

(19) $$G_{m}=y(a_{m},\;\theta )+P_{mm}$$

Non-Linear Regression centers on the concept of an expectation function, typically denoted by y. This function represents the predicted value of the dependent variable (often signifying the diabetic state) based on the independent variables (also known as features extracted from gene expression data) for each data point. Here is the key difference between non-linear and linear models: in Non-Linear Regression, at least one parameter ($a_{m}$) for each data point (m) must influence one or more derivatives of the expectation function.

### 5.2. Linear Regression

Linear Regression [51], a basis of statistical analysis, excels at modeling linear relationships between variables. However, in the realm of diabetes detection, it falls short as a primary classification tool, Linear Regression assumes a straight-line connection between the features extracted from gene expression data (independent variables) and the diabetic state (dependent variable). This linearity might not reflect the true complexities of the underlying biology. Diabetic development can involve intricate, non-linear relationships that Linear Regression might miss, leading to inaccurate classifications. Next, diabetes detection is inherently a binary classification problem. While applying a threshold might seem straightforward, it can be suboptimal for capturing the nuances of the data and the disease itself.

(20) Z=p+qX

### 5.3. Gaussian Mixture Models

Gaussian Mixture Models (GMMs) [52] shine as powerful tools for unsupervised machine learning. Unlike techniques requiring labeled data, GMMs excel at uncovering hidden structures within complex datasets like gene expression profiles. GMMs act like detectives, searching for hidden patterns in the data. They can automatically group similar gene expression profiles into clusters, potentially revealing subgroups within the diabetic or non-diabetic population. Unlike rigid clustering methods, GMMs employ a softer approach. They assign probabilities to each cluster for a given data point, allowing for potential overlap between clusters. This flexibility reflects the inherent complexities of diseases like diabetes, where gene expression patterns might not be perfectly distinct.

GMMs assume that the data are from a blend of multiple, simpler Gaussian distributions [53].

(21) $$p(a)=\frac{1}{{\left(2\pi \right)}^{\frac{n}{2}}{\left|\Sigma \right|}^{\frac{1}{2}}}e^{-\left(\frac{1}{2}\right){\left(a-\mu \right)}^{T}\Sigma^{-1}\left(a-\mu \right)}$$

Within the Gaussian distribution formula, the symbol μ denotes the mean vector, representing the average position of the data points in the n-dimensional space. Σ represents the covariance matrix, which captures the relationships between the different features (dimensions) within the data. The determinant of the covariance matrix, denoted by |Σ|, plays a role in calculating the overall spread of the data around the mean. Finally, the exp() function is used to calculate the probability density of a particular data point within the Gaussian distribution.

By uncovering hidden structures and capturing the complexities of gene expression data, GMMs provide valuable insights for researchers exploring novel approaches to diabetes detection.

### 5.4. Expectation Maximum

The EM algorithm emerges as a powerful tool for addressing missing information or hidden factors [54,55]. The EM algorithm acts like a skilled detective in diabetes detection. Two-step process such as:

- Expectation Step: In this initial step, the EM algorithm estimates the missing information, or hidden factors based on the currently available data and the current model parameters.
- Maximization Step: With the estimated missing values in place, the EM algorithm then refines the model parameters by considering the newly completed data.

**Figure 7:**
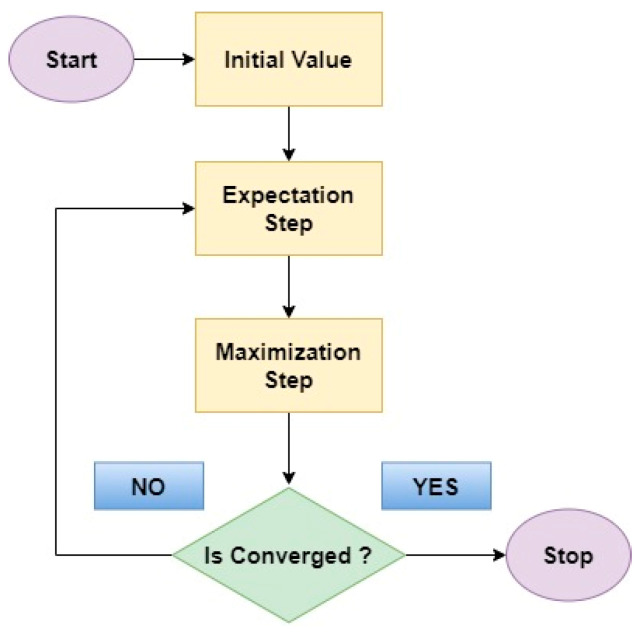
Flow diagram of EM algorithm.

Figure 7 represents the flow diagram of EM algorithm. Through this iterative process of estimating missing information and then improving the model based on those estimates, the EM algorithm gradually uncovers the underlying structure and parameters governing the distribution of gene expression data. This ultimately leads to a more accurate classification of diabetic and non-diabetic cases.

### 5.5. Logistic Regression

LR establishes itself as a powerful tool for binary classification tasks, proving its worth in decoding the complexities of diabetes detection. Like its role in identifying alcoholic tendencies, Logistic Regression [56] estimates the probability of an individual developing diabetes based on a collection of factors. The initial phase involves meticulously gathering relevant data on potential risk factors from a sample population. The collected data are then strategically divided into two distinct sets. The training set serves as the foundation for building the Logistic Regression model, while the testing set acts as an impartial evaluator, assessing the model’s accuracy in predicting new diabetic cases. The LR model [57] meticulously analyzes the training data. Once the model is fully trained and refined, it can be leveraged to predict the probability of diabetes in new individuals based solely on their specific risk factors.

(22) $$Logit\left\{\Pi \left(x\right)\right\}=\upsilon_{0}+\sum_{j=1}^{q}\upsilon_{j}x_{j}^{*}$$

The objective is to optimize both the fitness and log-likelihood, which can be achieved by attaining the subsequent function:

(23) $$1\left(\upsilon_{0},\upsilon \right)=\sum_{j=1}^{n}\left\{y_{i}\log\left(\pi_{i}\right)+\left(1-\pi_{i}\right)\right\}-\frac{1}{{2\tau }^{2}}{\left\|\upsilon \right\|}^{2}$$

### 5.6. Softmax Discriminant Classifier

SDC is an ingenious approach that leverages the power of an individual’s genetic makeup to predict their potential diabetes risk. SDC [58] is meticulously analyzing a person’s genes. It employs a specialized tool—a discriminant function—that acts like a comparison chart. This function compares the genetic profile against those of individuals confirmed to have or not have diabetes. The key strength of SDC lies in its ability to learn and adapt. The goal is to establish clear boundaries between these two groups, enabling accurate classification of new cases. When presented with a new genetic profile, SDC compares it to the learned patterns. By analyzing the closest match, SDC can estimate the likelihood of an individual developing diabetes.

The process of transforming class samples and test samples in SDC incorporates non-linear enhancement values, which are calculated utilizing the subsequent equations:

(24) $$\;h\left(K\right)=\arg\max Z_{w}^{i}$$

(25) $$h\left(K\right)=\arg\underset{i}{\max}\log\left(\sum_{j=1}^{d_{i}}\exp\left(-\lambda {\left\|v-\upsilon_{j}^{i}\right\|}_{2}\right)\right)$$

In these formulas, $h\left(K\right)$ represents the distinction of the i-th class, and as ${\left\|v-\upsilon_{j}^{i}\right\|}_{2}$ converges to zero, $Z_{w}^{i}$ is maximized. This characteristic ensures that the test sample is most likely to belong to a particular class.

### 5.7. Support Vector Machine (Radial Basis Function)

Support Vector Machines (SVMs) are a powerful machine learning technique well suited for classification tasks like diabetes detection. Unlike Linear Regression, SVMs can handle non-linear relationships between features extracted from gene expression data and the diabetic state. SVMs [59] can map the extracted features of initially existing in a lower-dimensional space into a higher-dimensional space. This transformation allows the data points to be separated more effectively using a hyperplane, even if the original relationship between features and the diabetic state was non-linear. RBF kernel is a popular choice for SVM classification. It acts like a bridge, calculating the similarity between data points in the higher-dimensional space. This similarity measure is then used by the SVM algorithm to identify the optimal hyperplane that best separates diabetic from non-diabetic data points in this transformed space. Once the optimal hyperplane is established, new, unseen data points can be mapped into the same higher-dimensional space using the RBF kernel. Their position relative to the hyperplane allows the SVM to classify them as diabetic or non-diabetic.

(26) $$\mathrm{RBF}:\;k\left(x_{i},x_{j}\right)=\exp\left\{\frac{-{\left|x_{i}-x_{j}\right|}^{2}}{{\left(2\times \sigma \right)}^{2}}\right\}$$

By effectively handling non-linearities and leveraging the RBF kernel for similarity calculations, SVMs offer a robust approach to classifying diabetic cases based on gene expression data.

### 5.8. Selection of Classifiers Parameters through Training and Testing

To ensure the model’s generalizability despite limited data, a robust technique called 10-fold cross-validation is employed. This method splits the data into 10 equal folds, trains the model on 9 folds, and tests it on the remaining fold. This process is repeated for all folds, providing a more reliable estimate of the model’s performance on unseen data compared to a single test/train split. Additionally, the model was trained on a dataset containing 2870 features per patient for 20 diabetic and 50 non-diabetic individuals. This comprehensive training process, combined with k-fold cross-validation, strengthens the reliability of the findings. The training process is measured in the MSE as:

(27) $$MSE=\frac{1}{N}\sum_{j=1}^{N}{\left(O_{j}-T_{j}\right)}^{2}$$

$O_{j}$ is the actual value observed at a specific time, and $T_{j}$ is the target value the model predicts for that time.

## 6. Classifiers Training and Testing

Table 6 represents the confusion matrix for diabetes detection and the parameters are defined as follows:

TP (True Positive): Catches diabetic patients.
TN (True Negative): Identifies healthy people.
FP (False Positive): Mistakes a healthy person for diabetic.
FN (False Negative): Misses a diabetic person.

Table 7 directly compares the Mean Squared Error (MSE) on both the training data it learns from (Train MSE) and unseen test data (Test MSE), with lower MSE indicating better performance. The highest values achieved as Support Vector Machine (SVM) with an RBF kernel, achieving exceptionally low MSE on both training and test data (Train MSE: 1.88 × 10^−6^, Test MSE: 1 × 10^−6^). Statistical models, like Ridge Regression (Train MSE: 7.29 × 10^−6^, Test MSE: 3.25 × 10^−5^), Linear Regression (Train MSE: 1.16 × 10^−5^, Test MSE: 1.94 × 10^−5^), and Logistic Regression with L1 penalty (LoR) (Train MSE: 2.7 × 10^−5^, Test MSE: 3.02 × 10^−5^) also show promise with consistently low MSE values. Mixture models, Gaussian Mixture Models (GMMs) (Train MSE: 1.02 × 10^−5^, Test MSE: 1.48 × 10^−5^) and Expectation Maximization (EM) (Train MSE: 5.29 × 10^−6^, Test MSE: 1.37 × 10^−5^), demonstrate competitive results. Notably, STFT and PCC have higher MSE values across both Train and Test data, suggesting they may not be as effective for this specific diabetes detection task. Note that all models exhibit lower MSE on the training data compared to test data, indicating some degree of overfitting. This underscores the importance of evaluating models using unseen test data to ensure they generalize well to new cases.

In Table 8, SVM with an RBF kernel maintains its dominant position (Train MSE: 2.18 × 10^−6^, Test MSE: 1.44 × 10^−6^). It achieves exceptionally low MSE on both the training data it learns from and unseen test data. Statistical models show mixed results. Ridge Regression (Train MSE: 1.44 × 10^−5^, Test MSE: 2.21 × 10^−5^) performs consistently, while LR (Train MSE: 3.76 × 10^−5^, Test MSE: 2.3 × 10^−5^) delivers slightly higher training MSE but achieves a lower test MSE. Logistic Regression with L1 penalty (Train MSE: 9.97 × 10^−6^, Test MSE: 1.76 × 10^−5^) demonstrates the most significant improvement in test MSE compared to training MSE. Mixture models present a similar picture. The GMM exhibits a significant jump in Test MSE (3.97 × 10^−4^) compared to Train MSE (4.51 × 10^−5^), suggesting potential overfitting. EM shows more balanced performance (Train MSE: 3.14 × 10^−4^, Test MSE: 1.37 × 10^−5^). Like the previous table, STFT and PCC remain less competitive in this task, evident from their higher MSE values across both Train and Test data (above 5.00×10^−5^ for most cases). An interesting observation is the significant reduction in Test MSE for SDC (Softmax Discriminant Classifier) compared to the previous table (Train MSE: 2.21 × 10^−5^, Test MSE: 1.6 × 10^−5^ to Train MSE: 2.81 × 10^−6^, Test MSE: 2.81 × 10^−4^). This suggests potential improvements in model generalizability. Overall, SVM with RBF kernel remains the leader, while statistical and mixture models show varying effectiveness depending on the specific model and its ability to generalize to unseen data. The significant reduction in SDC’s Test MSE warrants further investigation into its potential for diabetes detection.

In Table 9, SVM with an RBF kernel maintains its exceptional performance (Train MSE: 4.25 × 10^−7^, Test MSE: 3.6 × 10^−7^). It achieves remarkably low MSE on both the training and unseen test data, solidifying its position as the leading contender. Statistical models show further improvement. LoR (Train MSE: 4.85 × 10^−5^, Test MSE: 1.96 × 10^−6^) demonstrates a significant decrease in Test MSE compared to the previous table. Logistic Regression with L1 penalty (LoR) (Train MSE: 1.39 × 10^−5^, Test MSE: 2.25 × 10^−6^) also exhibits a positive trend with lower Test MSE. However, RR (Train MSE: 6.08 × 10^−5^, Test MSE: 9 × 10^−6^) shows a slight increase in Test MSE. Mixture models present a more promising picture in this table. The GMM achieves a significant reduction in both Train MSE (9.01 × 10^−6^) and Test MSE (4.41 × 10^−6^), suggesting potential improvements in addressing overfitting. EM maintains consistent performance (Train MSE: 3.51 × 10^−5^, Test MSE: 7.29 × 10^−6^). Like previous observations, STFT and PCC remain less competitive (Train and Test MSE values above 5.00 × 10^−6^). The most significant improvement is seen with SDC. It achieves exceptionally low Test MSE (1.96 × 10^−6^) compared to both previous tables (Train MSE: 1.35 × 10^−5^, Test MSE: 2.89 × 10^−6^). This dramatic reduction in Test MSE warrants further investigation into the potential of SDC for diabetes detection, especially considering its consistent Train MSE across all three tables.

Overall, SVM with RBF kernel remains the frontrunner. Statistical and mixture models demonstrate ongoing optimization, with Logistic Regression and GMMs showing the most notable improvements. The remarkable performance boost of SDC in this table highlights its potential as a viable approach for diabetes detection.

### Selection of Targets

The target value for the non-diabetic class ($T_{Non-Dia}$) ranges from 0 to 1, with emphasis on the lower end of the scale. This range is determined by a specific constraint [60].

$$\frac{1}{N}\sum_{i=1}^{N}\mu_{i}\leq T_{Non-Dia}$$

Here, $\mu_{i}$ denotes the mean value of the input feature vectors considered for classification across N non-diabetic features. Similarly, in the case of the diabetic class ($T_{Dia}$), the target value is aligned with the upper range of the 0 to 1 scale. This alignment is determined by the following principle as:

$$\frac{1}{M}\sum_{j=1}^{N}\mu_{j}\leq T_{Dia}$$

In this context, $\mu_{j}$ represents the average value of input feature vectors for the M diabetic cases considered in classification. It is crucial to note that the target value $(T_{Dia}$) is deliberately set higher than the average values of both $\mu_{i}$ and $\mu_{j}$. This choice of target values mandates a minimum discrepancy between them of 0.5, as stated below,

$$||T_{Dia}-T_{Non-Dia}||\geq 0.5$$

The target values for non-diabetic ($T_{Non-Dia}$) and diabetic ($T_{Dia}$) cases are set at 0.1 and 0.85, respectively. After setting the targets, MSE is employed to assess classifier performance. Table 10 illustrates the optimal parameter selection for the classifiers following the training and testing phases.

## 7. Outcomes and Findings

The evaluation approach ensures robust assessment of the models’ effectiveness in classifying diabetic patients. Researchers employ a widely used technique called “tenfold cross-validation”. Here, the data are meticulously divided into 10 equal sets. Nine of these sets (representing 90% of the data) are used to train the models. The remaining set (10%) serves a crucial purpose—testing the model’s performance on unseen data. This approach helps mitigate overfitting and provides a more reliable picture of how the models would perform in real-world scenarios. To go beyond simple accuracy, we leverage a valuable tool called a confusion matrix. This matrix allows us to calculate a comprehensive set of performance metrics. These metrics include accuracy (overall percentage of correct predictions), F1 score (balances precision and recall), MCC (considers true positives/negatives for a more robust evaluation), Jaccard Metric (measures the shared proportion of positive predictions between the model and the ground truth), Error Rate (percentage of incorrect predictions), and Kappa statistic [61] (measures agreement beyond chance). By analyzing these metrics derived from the confusion matrix, we gain a deeper understanding of the models’ strengths and weaknesses in distinguishing between diabetic and non-diabetic cases. Table 11 shows which methods are employed to calculate the performance metrics mentioned. This table provides a transparent view of the evaluation process and allows for further analysis of the models’ configuration.

The result of the above metrics for evaluation of the performance in each classifier is given in Table 12.

From Table 12, SVM with an RBF kernel solidified its dominance. It achieved the highest overall accuracy (92.8571%), F1 score (85.71%), Matthews Correlation Coefficient (MCC) (0.7979), and Jaccard similarity (75%), demonstrating its exceptional ability to distinguish between diabetic and non-diabetic cases With PCC as FE. Conversely, RR exhibited the lowest performance across most metrics, suggesting that it may not be suitable for this task. While the choice of feature extraction technique appears less impactful than the model itself (except for PCC), some interesting observations emerged. The combination of STFT and RR yielded the lowest overall performance (average accuracy of 80%), indicating RR might be less effective here. Excluding these outliers (SVM and Pearson CC), the average accuracy for other models ranged from 81% to 84%, with F1 scores between 72% and 74% and MCC values between 0.60 and 0.65. Overall, SVM with an RBF kernel emerged as the strongest contender for diabetes detection due to its consistent performance across various feature extraction techniques. While other models like NLR and SDC showed promise, further investigation might be necessary to evaluate their generalizability and confirm their effectiveness.

**Figure 8:**
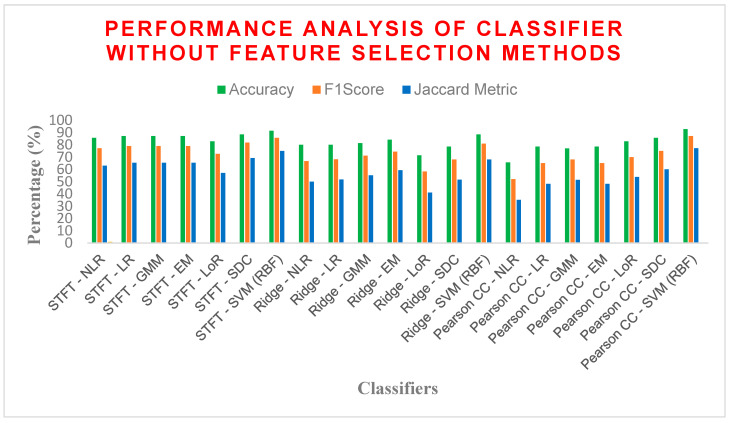
Performance analysis of different classifiers without the FS method.

From Figure 8, SVM with an RBF kernel consistently achieved the highest values across all performance metrics, demonstrating its exceptional ability to distinguish diabetic from non-diabetic cases. For instance, we observed exceptional accuracy (approximately 85%) and F1 score (approximately 80%) for SVM with RBF compared to other models. While the choice of FE technique seems to have a lower impact on overall performance compared to the model itself (except for RR), one noteworthy observation emerged. RR, when combined with any feature extraction technique, yielded the lowest performance. Excluding RR and the outliers (SVM and Pearson CC), other models exhibited average accuracy ranging from 80% to 85%, with F1 scores between 70% and 75%. Overall, SVM with RBF kernel stands out as the most promising approach for diabetes detection based on its consistently high performance across various feature extraction techniques. While other models like NLR, SDC, and some with other feature extraction techniques show promise, further investigation might be necessary to evaluate their generalizability and confirm their effectiveness.

From Table 13, SVM with RBF kernel solidified its dominance. It achieved the highest overall accuracy (92.86%), F1 score (87.80%), Matthews Correlation Coefficient (MCC) (0.8280), and Jaccard similarity (78.26%), demonstrating its exceptional ability to distinguish between diabetic and non-diabetic cases with PCC as FE for BESO FS. Conversely, PCC with NLR exhibited the lowest performance across most metrics, with accuracy as low as 57.14%, a F1 score of 44.44%, and a MCC of 0.1446. Examining the impact of FE techniques revealed some interesting insights. The pairing of STFT with RR yielded a moderate average accuracy of 78%, indicating RR might be less effective here. However, excluding RR techniques, with STFT in NLR, LoR, GMMs, EM achieved similar average accuracy (ranging from 80% to 84%) when combined with SVM (RBF), as high as 91.42%. This highlights the consistent performance of SVM (RBF) across various FE methods. In conclusion, SVM with an RBF kernel emerged as the strongest contender for diabetes detection based on its consistently high performance across different FE techniques.

**Figure 9:**
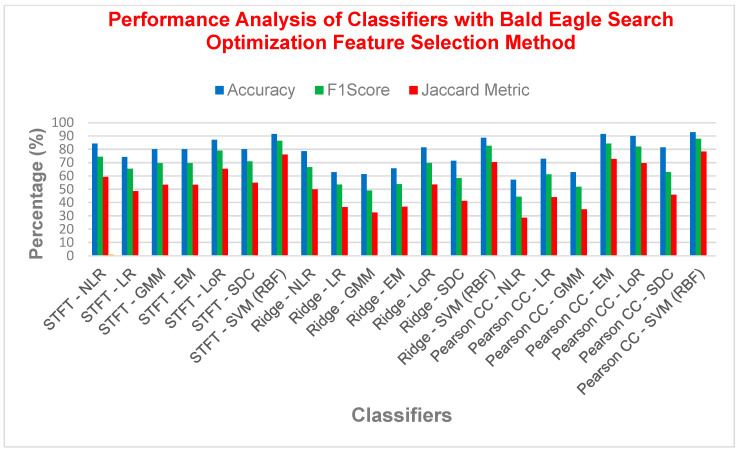
Performance analysis of different classifier with the Bald Eagle Search Optimization FS method.

Figure 9 revealed SVM with RBF kernel as the strongest classifier for diabetes detection, achieving exceptional performance (accuracy > 92%, F1 score > 87%, MCC > 0.82, Jaccard > 78%). Interestingly, the choice of feature extraction technique had less impact, except for Ridge Regression, which yielded the lowest performance across all techniques. While other models like NLR, LR, GMMs, EM, and SDC showed promise, SVM with RBF kernel emerged as the leader due to its consistent effectiveness.

From Table 14, it is observed that the diabetes detection models revealed SVM with RBF kernel as the champion, achieving exceptional results (accuracy reached the highest ever as 97.14%, F1 score reaches 95%, MCC exceeds 0.93, Jaccard exceeds 90%). This dominance is evident compared to the lowest performing model, Pearson CC with NLR, which obtained an accuracy as low as 62%, a F1 score of 48%, and a MCC of 0.22. Excluding these outliers, the average performance across models ranged from 80% to 85% accuracy, 70% to 78% F1 score, and 0.55 to 0.65 MCC. Interestingly, the choice of feature extraction technique seems to have a lower impact on performance, except for Ridge Regression. It consistently yielded the lowest values (accuracy approximately 65%, F1 score approximately 55%, MCC approximately 0.30) regardless of the technique used. Techniques like NLR, LR, GMMs, EM, LoR, and SDC performed similarly well with SVM (RBF), all achieving average accuracy above 80%. This highlights the robustness of SVM with RBF across various feature extraction approaches.

**Figure 10:**
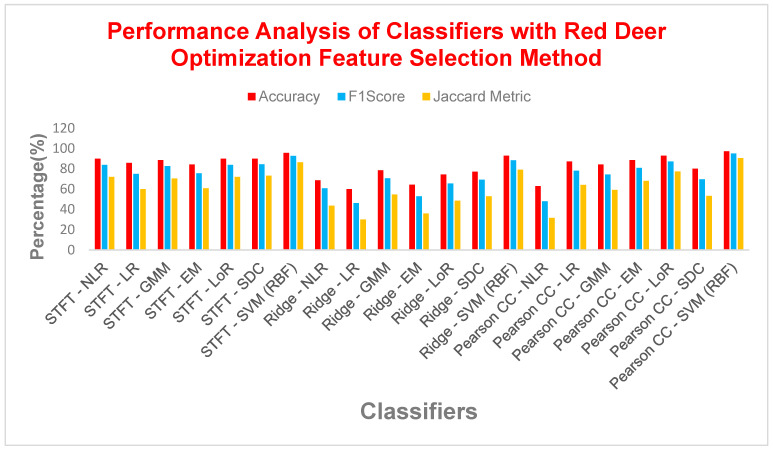
Performance analysis of various classifiers with the Red Deer Optimization FS method.

From Figure 10, it is observed that from the analysis of diabetes detection models, SVM with RBF kernel emerged as the clear leader, achieving over 97% accuracy, with F1 score exceeding 95% and MCC surpassing 0.93. Interestingly, the choice of feature extraction method had minimal impact on performance, as models like NLR, LR, GMMs, EM, LoR, and SDC performed similarly well with SVM (RBF), all achieving an average accuracy of over 80%. However, Ridge Regression consistently underperformed across all metrics, suggesting that it may not be the best choice for this task. Overall, SVM with RBF kernel demonstrated exceptional and consistent performance, making it the top contender for diabetes detection.

**Figure 11:**
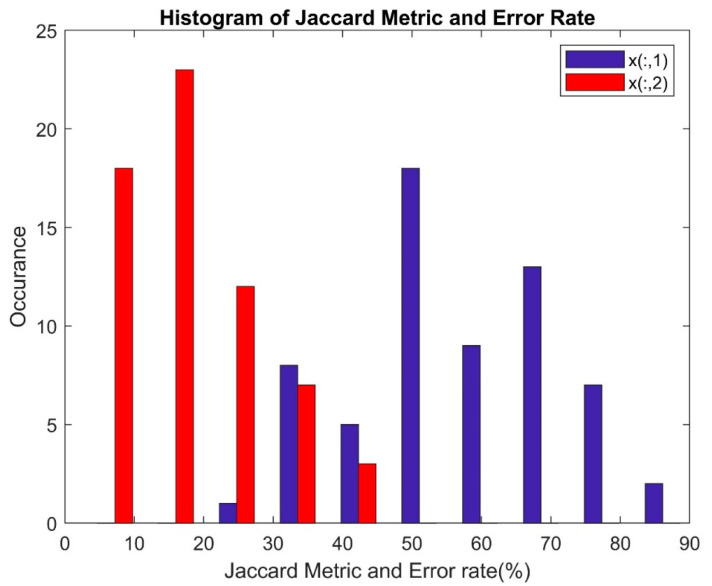
Performance of Jaccard Metric and Error Rate.

Figure 11 illustrates the performance of the Jaccard Metric and Error Rate (%) parameters through histograms. It is observed that the maximum Error Rate stabilizes at 50%, while the maximum Jaccard Metric reaches 90%. The histogram representing the Error Rate is skewed towards the right side of the graph, indicating that regardless of the feature extraction method or feature selection method employed, the classifier’s Error Rate remains below 50%. On the other hand, the histogram of the Jaccard Metric displays sparsity towards the edges and covers a greater number of points in the central area across the classifier.

### 7.1. Computational Complexity

This study evaluates classifiers based on their Computational Complexity (CC), specifically focusing on how it scales with the size of the input data (denoted as O(n)). Ideally, a classifier should have a low CC, represented as O(1). This indicates that the algorithm’s complexity remains constant regardless of how much data it needs to process. This is a desirable characteristic because it ensures efficient performance even with large datasets. Interestingly, the analysis highlights that the CC in these models is independent of the input size, further emphasizing their efficiency. However, the text also mentions that some models exhibit a logarithmic complexity (O(log n)) as the data size increases. Additionally, the study explores hybrid models that incorporate DR techniques and feature selection methods within their classification process. These techniques can potentially influence the CC of the overall model.

Table 15 reveals a range of complexities across the classifiers. NLR, LR, LoR, and SDC show a complexity of O(n^2^ logn) during training and then increase to O(2n^2^ log2n) or O(2n^2^ logn) for prediction. GMMs and EM are slightly more complex, reaching O(n^2^ log2n) in training and O(2n^3^ log2n) for prediction. However, SVM (RBF) stands out as the most computationally demanding, reaching O(2n^4^ log2n) during training and a slightly lower O(2n^2^ log4n) for prediction. In conclusion, the chosen classifier and FE technique significantly impact the computational cost of your analysis. While all classifiers show increased complexity due to the FE techniques, SVM (RBF) is the most demanding. For large datasets and a balance between efficiency and potentially good performance, consider classifiers like NLR, LR, or LoR with these FE techniques.

Table 16 reveals a range of complexities. NLR, LR, and LoR show a complexity of O(n^4^ logn) during training and then increase to O(2n^4^ log2n) for prediction. GMMs and EM are slightly more complex, reaching O(n^4^ log2n) in training and O(2n^5^ log2n) for prediction. However, SVM (RBF) stands out as the most demanding, reaching a staggering O(2n^6^ log2n) during training and a slightly lower O(2n^4^ log4n) for prediction. In conclusion, both the chosen classifier and FE technique significantly impact the computational cost of your analysis. While all classifiers show increased complexity due to the FE techniques, SVM (RBF) is the most challenging.

Table 17 reveals the impact of FE techniques, such as STFT, Ridge Regression, and Pearson CC, on overall complexity with RDO FS techniques. These techniques introduce additional computations, leading to a notable increase in CC across all classifiers. The table provided illustrates a spectrum of complexities for classifiers employed with these FE techniques. NLR, LR, and LoR exhibit a complexity of O(n^5^ logn), while GMMs and EM are slightly more complex, reaching O(n^5^ log2n). SDC demonstrates a complexity of O(n^6^ logn). However, SVM (RBF) stands out as the most demanding, with a staggering complexity of O(2n^7^ log2n). Regarding feature selection, it is crucial to note that this analysis solely focuses on CC based on the chosen FE techniques. Techniques like RDO for feature selection have the potential to reduce the number of features utilized in the classification process, significantly lowering the overall complexity of the model compared to using all features.

### 7.2. Limitations

Table 18 compares the results are various machine learning methods in terms of accuracy prediction. Investigated the potential of using microarray gene data to identify type 2 diabetes early and potentially predict associated diseases. The analysis revealed promising classification techniques that could be valuable for screening and identifying diabetes markers, along with potentially linked diseases like strokes and kidney problems. While the findings may be specific to this patient group and require further validation, they pave the way for future research. The methods used, such as microarrays, might not be readily available in all settings due to cost and complexity. However, this study lays the groundwork for developing more accessible and efficient approaches for early diabetes detection and disease management. Overall, this research highlights the potential for early detection of type 2 diabetes and associated diseases, emphasizing the importance of further research to validate these findings and develop more accessible screening methods for improved patient outcomes.

### 7.3. Conclusions and Future Work

This analysis explored various FE techniques (STFT, Ridge Regression, and PCC) and feature selection methods (BESO and RDO) for their impact on classifier performance in detecting Type II DM using microarray gene data. While RR with RDO FS yielded lower accuracy, STFT and PCC showcased improved performance metrics, particularly for the SVM (RBF) classifier. Notably, the combination of SVM (RBF) and RDO achieved the highest accuracy (92.85%) even without feature selection. However, employing RDO alongside SVM (RBF) still resulted in the highest accuracy across all FE techniques (95%, 92%, and 97.14% for STFT, RR, and PCC, respectively). BESO also yielded promising results, achieving high accuracy values (approximately 90%) with all three FE techniques. Interestingly, Computational Complexity remained similar across classifiers with different dimensionality reduction methods, highlighting the crucial role of feature selection in boosting accuracy. In conclusion, this study presents a novel approach for Type II DM detection using microarrays. Future research will explore the application of Convolutional Neural Networks (CNNs), Deep Learning Networks (DNNs), and Long Short-Term Memory (LSTM) networks, along with hyperparameter tuning for further performance optimization.

## Figures and Tables

**Table 1:** Review of previous work.

S. No.	Author and Year	Database	Feature Extraction/Dimensionality Reduction Technique	Classifiers Used	Evaluation Metrics	Limitations
1	Kumar, D. A., and Govindasamy, R. (2015) [18]	UCI repository	-	Support Vector Regression Bayes Net Naive Bayes Decision Table	79.81% accuracy	The study only used the Pima Indians Diabetes Dataset (PIDD) from the UCI Machine Learning Repository, which may limit the generalizability of the findings to other diabetes datasets or real-world scenarios.
2	Lawi, A. and Syarif, S. (2019, October) [19]	GSE18732 Mircoarray gene expression data	Entropy and Resampling (SMOTE)	Naïve Bayes, SVM: Linear, RBF, Polynomial	SVM uses RBF kernel achieved 97.22% accuracy	The limited dataset size of only 2000 customer reviews, which may not be sufficient to fully evaluate the performance of the Naive Bayes and SVM classifiers.
3	Jakka et al. (2019) [20]	PIMA Dataset	-	KNN—K-Nearest Neighbor DT—Decision Tree Naive Bayes SVM LR—Logistic Regression RF—Random Forest	Accuracy: 73, 70, 75, 66, 78, 74	Potentially remove irrelevant or redundant features from the dataset.
4	Yang et al. (2020) [21]	NHANES (National Health and Nutrition Examination Survey) database	Binary Logistic Regression	Linear discriminant analysis, Support Vector Machine Random Forest	Accuracy: 75, 74, 74	The inability to clearly separate type 1 and type 2 diabetes, the unbalanced dataset, and the relatively low positive predictive value of the models.
5	Marateb et al. (2014) [22]	Tested on a sample size of 200 patients with type 2 diabetes in a cross-sectional study	Multimethods (machine learning, fuzzy logic, expert system)	SVM DT NB	Accuracy = 92%, 89%, 85%	Without a larger-scale validation, the true capabilities and limitations of the proposed hybrid intelligent system for diagnosing microalbuminuria remain uncertain.
6	Huang et al. (2015) [23]	Clinical and genotyping data study involving 345 type 2 diabetic patients (185 with diabetic nephropathy and 160 without diabetic nephropathy)	Clinical + Genetic Analysis	DT RF NB SVM	For DT—accuracy = 65.2% sensitivity = 63.2% specificity = 67.2%	A larger and more diverse dataset would be needed to further validate and refine the proposed Decision Tree-based model to identify diabetic nephropathy.
7	Chikh et al. (2012) [24]	UCI machine learning repository	Artificial Immune Recognition System	KNN CRISP Fuzzy	Accuracy = 89.10%	The article does not provide a detailed comparison of the MAIRS2 method with other commonly used machine learning techniques for diabetes diagnosis.
8	Luo, G. (2016) [25]	Electronic medical record dataset from the Practice Fusion diabetes classification competition containing patient records from all 50 states in the United States	Champion machine learning model	SVM	AUC = 0.884 Accuracy = 77.6%	Lack of model interpretability.
9	Kim et al. (2021) [26]	Aged 40–69 years from the combined dataset of the 4th to 7th KNHANES (from 2007 to 2018)	-	Deep Neural Network, logistic regression, Decision Tree	Accuracy: 80%, 80%, 71%	The study lacks detailed discussion on the specific parameters and variables used in the Deep Neural Network (DNN) model.
10	Kalagotla et al. (2021) [27]	PIMA Dataset		Stacking multilayer perceptron, Support Vector Machine Logistic Regression	Accuracy: 78 Precision: 72 Sensitivity: 51 F1 score: 60	The study did not explore the impact of different hyperparameters or feature engineering techniques on the performance of the proposed models, which could influence the overall results.
11	Sarwar et al. (2018) [28]	Pima Indians Diabetes Dataset	-	K-Nearest Neighbors (KNN), Naive Bayes (NB), Support Vector Machine (SVM), Decision Tree (DT), Logistic Regression (LR) and Random Forest (RF)	Accuracy: 0.77, 0.74, 0.77, 0.71, 0.74, 0.71	Lack of discussion on the potential biases present in the dataset used for predictive analytics.
12	Li et al. (2021) [29]	1512 subjects were recruited from the hospital	Genetic Algorithm	Extreme gradient boosting (GBT)	AUC (ROC): 0.91 precision: 0.82 sensitivity: 0.80 F1 score: 0.77	Limits the generalizability of the models, especially the deep learning models which typically require large datasets to perform optimally.

**Table 2:** Details of the dataset before and after feature extraction and feature selection.

Dataset Details	Status of the Data	Total No of Genes (per Patients)	After Feature Extraction—For All Three Methods (per Patients)	After Feature Selection—For All Two Methods (per Patients)
Nordic Islet Transplantation Program	Imbalanced Diabetic—20 Non-Diabetic—57	28,960	2870 × 1	287 × 1

**Table 3:** Statistical analysis for different feature extraction techniques.

Statistical Parameters	STFT		Ridge Regression		Pearson CC	
	Dia P	Non-Dia P	Dia P	Non-Dia P	Dia P	Non-Dia P
Mean	40.7681	40.7863	0.0033	0.0025	0.0047	0.0045
Variance	11,745.67	11,789.27	1.3511	1.3746	0.0004	0.0004
Skewness	19.2455	19.2461	0.0284	−0.0032	0.0038	−0.0317
Kurtosis	388.5211	388.5372	0.6909	0.9046	−0.1658	−0.0884
Sample Entropy	11.0014	11.0014	11.4868	11.4868	11.4868	11.4868
Shannon Entropy	0	0	3.9818	3.9684	2.8979	2.9848
Higuchi’s Fractal Dimension	1.1097	1.1104	2.007	2.0093	1.9834	1.9659
CCA	0.4031		0.0675		0.0908	

**Table 4:** Parameters of RDO.

S. No.	Parameters	Values	S. No.	Parameters	Values
1.	Initial population (I)	100	6.	Beta (β)	0.4
2.	Maximum time of simulation	10 (s)	7.	Gamma (γ)	0.7
3.	Number of males (M)	15	8.	Roar	0.25
4.	Number of hinds (H)	I − M	9.	Fight	0.4
5.	Alpha (α)	0.85	10.	Mating	0.77

**Table 5:** Utilizing *p*-values for feature selection in diabetes detection: A comparison of various FE techniques.

Feature Selection	DR Techniques	STFT		Ridge Regression		Pearson CC	
	Class	Dia P	Non-Dia P	Dia P	Non-Dia P	Dia P	Non-Dia P
BESO	*p* value < 0.05	0.4673	0.3545	0.2962	0.2599	0.3373	0.3178
RDO	*p* value < 0.05	0.4996	0.4999	0.4999	0.4883	0.4999	0.4999

**Table 6:** Confusion matrix of diabetic and non-diabetic classification.

Clinical Situation		Predicted Values	
		Dia	Non-Dia
Real Values	Class of Dia	TP	FN
	Class of Non-Dia	FP	TN

**Table 7:** Mean Squared Error analysis for different FE techniques without feature selection.

Classifiers	STFT		Ridge Regression		Pearson CC	
	Train MSE	Test MSE	Train MSE	Test MSE	Train MSE	Test MSE
NLR	1.59 × 10^−5^	4.84 × 10^−6^	7.29 × 10^−6^	3.25 × 10^−5^	4.36 × 10^−5^	4.1 × 10^−4^
LR	1.18 × 10^−5^	3.61 × 10^−6^	1.16 × 10^−5^	1.94 × 10^−5^	9.61 × 10^−6^	3.84 × 10^−4^
GMM	1.05 × 10^−5^	2.89 × 10^−6^	1.02 × 10^−5^	1.48 × 10^−5^	2.02 × 10^−5^	8.41 × 10^−4^
EM	6.74 × 10^−6^	2.89 × 10^−6^	5.29 × 10^−6^	1.37 × 10^−5^	9.61 × 10^−6^	3.72 × 10^−5^
LoR	2.46 × 10^−5^	9 × 10^−6^	2.7 × 10^−5^	3.02 × 10^−5^	4 × 10^−6^	2.92 × 10^−5^
SDC	1.28 × 10^−5^	4 × 10^−6^	1.68 × 10^−5^	1.22 × 10^−5^	2.56 × 10^−6^	1.85 × 10^−5^
SVM (RBF)	1.88 × 10^−6^	1 × 10^−6^	2.56 × 10^−6^	4.41 × 10^−6^	3.6 × 10^−7^	4.41 × 10^−6^

**Table 8:** Mean Squared Error analysis for different feature extraction techniques with Bald Eagle Search Optimization (BESO) feature selection.

Classifiers	STFT		Ridge Regression		Pearson CC	
	Train MSE	Test MSE	Train MSE	Test MSE	Train MSE	Test MSE
NLR	1.43 × 10^−5^	5.29 × 10^−5^	1.44 × 10^−5^	2.21 × 10^−5^	9.41 × 10^−5^	7.06 × 10^−5^
LR	3.76 × 10^−5^	2.3 × 10^−5^	7.74 × 10^−5^	1.85 × 10^−5^	2.5 × 10^−5^	2.02 × 10^−5^
GMM	4.51 × 10^−5^	1.3 × 10^−5^	6.56 × 10^−5^	3.97 × 10−4	6.08 × 10^−5^	3.02 × 10^−5^
EM	3.4 × 10^−5^	1.37 × 10^−5^	5.18 × 10^−5^	3.14 × 10−4	1.6 × 10^−7^	1.3 × 10^−5^
LoR	9.97 × 10^−6^	4 × 10^−6^	9 × 10^−6^	1.76 × 10^−5^	4.9 × 10^−7^	1.68 × 10^−5^
SDC	2.21 × 10^−5^	1.6 × 10^−5^	2.81 × 10^−6^	2.81 × 10−4	8.1 × 10^−7^	8.65 × 10^−5^
SVM (RBF)	2.18 × 10^−6^	1.44 × 10^−6^	5.29 × 10^−6^	4.9 × 10^−5^	4.9 × 10^−7^	8.1 × 10^−7^

**Table 9:** Mean Squared Error analysis for different feature extraction techniques with Red Deer Optimization (RDO) feature selection.

Classifiers	STFT		Ridge Regression		Pearson CC	
	Train MSE	Test MSE	Train MSE	Test MSE	Train MSE	Test MSE
NLR	2.62 × 10^−5^	2.56 × 10^−6^	6.08 × 10^−5^	9 × 10^−6^	5.04 × 10^−5^	6.56 × 10^−5^
LR	4.85 × 10^−5^	1.96 × 10^−6^	6.24 × 10^−5^	6.4 × 10^−5^	2.25 × 10^−6^	1.09 × 10^−5^
GMM	9.01 × 10^−6^	4.41 × 10^−6^	2.12 × 10^−5^	2.25 × 10^−6^	6.25 × 10^−6^	1.22 × 10^−5^
EM	3.51 × 10^−5^	7.29 × 10^−6^	5.48 × 10^−5^	2.81 × 10^−5^	1.69 × 10^−6^	7.84 × 10^−6^
LoR	1.39 × 10^−5^	2.25 × 10^−6^	3.02 × 10^−5^	4.84 × 10^−6^	3.6 × 10^−7^	4 × 10^−6^
SDC	1.35 × 10^−5^	2.89 × 10^−6^	2.6 × 10^−5^	1.96 × 10^−6^	1.44 × 10^−7^	1.68 × 10^−5^
SVM (RBF)	4.25 × 10^−7^	3.6 × 10^−7^	8.1 × 10^−7^	9 × 10^−8^	4 × 10^−8^	2.5 × 10^−7^

**Table 10:** Classifiers optimal parameters.

Classifiers	Description
NLR	The uniform weight is set to 0.4, while the bias is adjusted iteratively to minimize the sum of least square errors, with the criterion being the Mean Squared Error (MSE).
Linear Regression	The weight is uniformly set at 0.451, while the bias is adjusted to 0.003 iteratively to meet the Mean Squared Error (MSE) criterion.
GMM	The input sample’s mean covariance and tuning parameter are refined through EM steps, with MSE as the criterion.
EM	The likelihood probability is 0.13, the cluster probability is 0.45, and the convergence rate is 0.631, with the condition being MSE.
Logistic Regression	The criterion is MSE, with the condition being that the threshold H*θ*(x) should be less than 0.48.
SDC	The parameter Γ is set to 0.5, alongside mean target values of 0.1 and 0.85 for each class.
SVM (RBF)	The settings include C as 1, the coefficient of the kernel function (gamma) as 100, class weights at 0.86, and the convergence criterion as MSE.

**Table 11:** Performance metrics.

Metrics	Formula
Accuracy	$Accu=\frac{\left(TN+TP\right)}{\left(TN+FN+TP+FP\right)}$
F1 Score	$F1=\frac{2\times TP}{\left(2\times TP+FP+FN\right)}$
Matthews Correlation Coefficient (MCC)	$MCC=\frac{\left(TP\times TN-FP\times FN\right)}{\sqrt{TP+FP)\times (TP+FN)\times (TN+FP)\times (TN+FN)}}$
Jaccard Metric	$Jacc=\frac{TP}{TP+FP+FN}$
Error Rate	ER = 1 − Accu
Kappa	$Kappa=\frac{{(P}_{o}-P_{e})}{\left(1-P_{e}\right)}$ $P_{o}=\frac{\left(TP+TN\right)}{\left(TP+TN+FP+FN\right)}$ $P_{e}=\frac{\left(TP+FP)\times (TP+FN)+(FP+TN)\times (FN+TN\right)}{{\left(TP+TN+FP+FN\right)}^{2}}$

**Table 12:** Analysis of different parameters with different classifiers through various FE techniques without feature selection techniques.

Feature Extraction	Classifiers	Parameters					
		Accu (%)	F1S (%)	MCC	Jaccard Metric (%)	Error Rate (%)	Kappa
STFT	NLR	85.7142	77.2727	0.6757	62.9629	14.2857	0.6698
	LR	87.1428	79.0697	0.7021	65.3846	12.8571	0.6985
	GMM	87.1428	79.0697	0.7021	65.3846	12.8571	0.6985
	EM	87.1428	79.0697	0.7021	65.3846	12.8571	0.6985
	LoR	82.8571	72.7272	0.6091	57.1428	17.1428	0.6037
	SDC	88.5714	81.8181	0.7423	69.2307	11.4285	0.7358
	SVM (RBF)	91.4285	85.7142	0.7979	75	8.57142	0.7961
Ridge Regression	NLR	80	66.6667	0.5255	50	20	0.5242
	LR	80	68.1818	0.5425	51.7241	20	0.5377
	GMM	81.4285	71.1111	0.5845	55.1724	18.5714	0.5767
	EM	84.2857	74.4186	0.6348	59.2592	15.7142	0.6315
	LoR	71.4286	58.3333	0.3873	41.1764	28.5714	0.375
	SDC	78.5714	68.0851	0.5383	51.6129	21.4285	0.5248
	SVM (RBF)	88.5714	80.9524	0.7298	68	11.4285	0.7281
Pearson CC	NLR	65.7143	52	0.2829	35.1351	34.2857	0.2695
	LR	78.5714	65.1162	0.5001	48.2758	21.4285	0.4976
	GMM	77.1429	68	0.5385	51.5151	22.8571	0.5130
	EM	78.5714	65.1162	0.5001	48.2758	21.4285	0.4976
	LoR	82.8571	70	0.58	53.8461	17.1428	0.58
	SDC	85.7142	75	0.65	60	14.2857	0.65
	SVM (RBF)	92.8571	87.1795	0.8228	77.2727	7.14285	0.8223

**Table 13:** Analysis of different parameters with different classifiers through various FE techniques with Bald Eagle Search Optimization feature selection techniques.

Feature Extraction	Classifiers	Parameters					
		Accu (%)	F1S (%)	MCC	Jaccard Metric (%)	Error Rate (%)	Kappa
STFT	NLR	84.2857	74.4186	0.6347	59.2592	15.7142	0.6315
	LR	74.2857	65.3846	0.4987	48.5714	25.7142	0.4661
	GMM	80	69.5652	0.5609	53.3333	20	0.5504
	EM	80	69.5652	0.5609	53.3333	20	0.5504
	LoR	87.1428	79.0697	0.7021	65.3846	12.8571	0.6985
	SDC	80	70.8333	0.5809	54.8387	20	0.5625
	SVM (RBF)	91.4285	86.3636	0.8089	76	8.57142	0.8018
Ridge Regression	NLR	78.5714	66.6667	0.5185	50	21.4285	0.5116
	LR	62.8571	53.5714	0.2982	36.5853	37.1428	0.2661
	GMM	61.4285	49.0566	0.2262	32.5	38.5714	0.2092
	EM	65.7142	53.8461	0.3083	36.8421	34.2857	0.2881
	LoR	81.4285	69.7674	0.5674	53.5714	18.5714	0.5645
	SDC	71.4285	58.3333	0.3872	41.1764	28.5714	0.375
	SVM (RBF)	88.5714	82.6087	0.7573	70.3703	11.4285	0.7431
Pearson CC	NLR	57.1428	44.4444	0.1446	28.5714	42.8571	0.1322
	LR	72.8571	61.2244	0.4310	44.1176	27.1428	0.4140
	GMM	62.8571	51.8518	0.2711	35	37.1428	0.2479
	EM	91.4285	84.2105	0.7855	72.7272	8.57142	0.7835
	LoR	90	82.0512	0.7517	69.5652	10	0.7512
	SDC	81.4285	62.8571	0.5174	45.8333	18.5714	0.5081
	SVM (RBF)	92.8571	87.8048	0.8280	78.2608	7.14285	0.8275

**Table 14:** Analysis of different parameters with different classifiers through various FE techniques with Red Deer Optimization feature selection techniques.

Feature Extraction	Classifiers	Parameters					
		Accu (%)	F1S (%)	MCC	Jaccard Metric (%)	Error Rate (%)	Kappa
STFT	NLR	90	83.7209	0.7694	72	10	0.76555
	LR	85.7142	75	0.65	60	14.2857	0.65
	GMM	88.5714	82.6087	0.7573	70.3703	11.4285	0.7431
	EM	84.2857	75.5555	0.6505	60.7142	15.7142	0.6418
	LoR	90	83.7209	0.7694	72	10	0.7655
	SDC	90	84.4444	0.7825	73.0769	10	0.7721
	SVM (RBF)	95.7142	92.6829	0.8971	86.3636	4.2857	0.8965
Ridge Regression	NLR	68.5714	60.7142	0.4248	43.5897	31.4285	0.3790
	LR	60	46.1538	0.1813	30	40	0.1694
	GMM	78.5714	70.5882	0.5820	54.5454	21.4285	0.5493
	EM	64.2857	52.8301	0.2895	35.8974	35.7142	0.2677
	LoR	74.2857	65.3846	0.4987	48.5714	25.7142	0.4661
	SDC	77.1428	69.2307	0.5622	52.9411	22.8571	0.5254
	SVM (RBF)	92.8571	88.3720	0.8367	79.1667	7.14285	0.8325
Pearson CC	NLR	62.8571	48	0.2190	31.5789	37.1428	0.2086
	LR	87.1428	78.0487	0.6901	64	12.8571	0.6896
	GMM	84.2857	74.4186	0.6347	59.2592	15.7142	0.6315
	EM	88.5714	80.9523	0.7298	68	11.4285	0.7281
	LoR	92.8571	87.1794	0.8228	77.2727	7.14285	0.8223
	SDC	80	69.5652	0.5609	53.3333	20	0.5504
	SVM (RBF)	97.1428	95	0.93	90.4761	2.85714	0.93

**Table 15:** Computational Complexity of different classifiers without feature selection.

Classifiers	DR Method		
	STFT	Ridge Regression	Pearson CC
NLR	O(n^2^ logn)	O(2n^2^ log2n)	O(2n^2^ log2n)
LR	O(n^2^ logn)	O(2n^2^log2n)	O(2n^2^ log2n)
GMM	O(n^2^ log2n)	O(2n^3^ log2n)	O(2n^3^ log2n)
EM	O(n^3^ logn)	O(2n^3^ log2n)	O(2n^3^ log2n)
LoR	O(2n^2^ logn)	O(2n^2^ log2n)	O(2n^2^ log2n)
SDC	O(n^3^ logn)	O(2n^2^ log2n)	O(2n^2^ log2n)
SVM (RBF)	O(2n^4^ log2n)	O(2n^2^ log4n)	O(2n^2^ log4n)

**Table 16:** Computational Complexity of different classifiers with BESO features selection.

Classifiers	DR Method		
	STFT	Ridge Regression	Pearson CC
NLR	O(n^4^ logn)	O(2n^4^ log2n)	O(2n^4^ log2n)
LR	O(n^4^ logn)	O(2n^4^ log2n)	O(2n^4^ log2n)
GMM	O(n^4^ log2n)	O(2n^5^ log2n)	O(2n^5^ log2n)
EM	O(n^5^ logn)	O(2n^5^ log2n)	O(2n^5^ log2n)
LoR	O(2n^4^ logn)	O(2n^4^ log2n)	O(2n^4^ log2n)
SDC	O(n^5^ logn)	O(2n^4^ log2n)	O(2n^4^ log2n)
SVM (RBF)	O(2n^6^ log2n)	O(2n^4^ log4n)	O(2n^4^ log4n)

**Table 17:** Computational Complexity of different classifiers with RDO features selection.

Classifiers	DR Method		
	STFT	Ridge Regression	Pearson CC
NLR	O(n^5^ logn)	O(2n^5^ log2n)	O(2n^5^ log2n)
LR	O(n^5^ logn)	O(2n^5^ log2n)	O(2n^5^ log2n)
GMM	O(n^5^ log2n)	O(2n^6^ log2n)	O(2n^6^ log2n)
EM	O(n^6^ logn)	O(2n^6^ log2n)	O(2n^6^ log2n)
LoR	O(2n^5^ logn)	O(2n^5^ log2n)	O(2n^5^ log2n)
SDC	O(n^6^ logn)	O(2n^5^ log2n)	O(2n^5^ log2n)
SVM (RBF)	O(2n^7^ log2n)	O(2n^5^ log4n)	O(2n^5^ log4n)

**Table 18:** Comparison with previous work.

S. No.	Author (with Year)	Description of the Population	Data Sampling	Machine Learning Parameter	Accuracy (%)
1.	This article	Nordic Islet Transplantation program	Tenfold cross-validation	STFT, RR, PCC, NLR, LR, LoR, GMM, EM, SDC, SVM (RBF)	97.14
2.	Maniruzzaman et al. (2017) [62]	PIDD (Pima Indian diabetic dataset)	Cross-validation K2, K4, K5, K10, and JK	LDA, QDA, NB, GPC, SVM, ANN, AB, LoR, DT, RF	ACC: 92
3.	Hertroijs et al. (2018) [63]	Total: 105,814 Age (mean): greater than 18	Training set of 90% and test set of 10% fivefold cross-validation	Latent Growth Mixture Modeling (LGMM)	ACC: 92.3
4.	Deo et al. (2019) [64]	Total: 140 diabetes: 14 imbalanced age: 12–90	Training set of 70% and 30% test set with fivefold cross-validation, holdout validation	BT, SVM (L)	ACC: 91
5.	Akula et al. (2019) [65]	PIDD Practice Fusion Dataset total: 10,000 age: 18–80	Training set: 800; test set: 10,000	KNN, SVM, DT, RF, GB, NN, NB	ACC: 86
6.	Xie et al. (2019) [66]	Total: 138,146 diabetes: 20,467 age: 30–80	Training set is approximately 67%, test set is approximately 33%	SVM, DT, LoR, RF, NN, NB	ACC: 81, 74, 81, 79, 82, 78
7.	Bernardini et al. (2020) [67]	Total: 252 diabetes: 252 age: 54–72	Tenfold cross-validation	Multiple instance learning boosting	ACC: 83
8.	Zhang et al. (2021) [68]	Total: 37,730, diabetes: 9.4% age: 50–70 imbalanced	Training set is approximately 80% test set is approximately 20% Tenfold cross-validation	Bagging boosting, GBT, RF, GBM	ACC: 82

## Data Availability

The data that support the findings of this study are available from the corresponding author upon reasonable request.
